# Prevalence of work-related musculoskeletal disorders among workers in the automobile manufacturing industry in China: a systematic review and meta-analysis

**DOI:** 10.1186/s12889-023-16896-x

**Published:** 2023-10-19

**Authors:** Xiongda He, Bin Xiao, Junle Wu, Chunshuo Chen, Wankang Li, Maosheng Yan

**Affiliations:** 1 School of Public Health, Southern Medical University, Guangzhou, Guangdong China; 2 Guangdong Province Hospital for Occupational Disease Prevention and Treatment, Guangzhou, Guangdong China; 3 School of Public Health, Guangzhou Medical University, Guangzhou, Guangdong China; 4 School of Public Health, Shanxi Medical University, Taiyuan, Shanxi China

**Keywords:** Automobile manufacturing, Musculoskeletal disorders, Chinese, Systematic review and Meta-analysis, Prevalence

## Abstract

**Objectives:**

Work-related musculoskeletal disorders (WMSDs) have become one of the major occupational health problems. Lots of auto workers in China are exposed to WMSDs. However, there are few systematic review and meta-analysis about WMSDs in this field. This study aimed to evaluate the prevalence of WMSDs among these workers.

**Methods:**

This study was carried out using the Systematic Reviews and Meta-analyses method under the most up to date PRISMA guidelines. A literature search was conducted for studies on the epidemiology of WMSDs among auto workers in China from inception to August 2022, using English and Chinese databases (China National Knowledge Infrastructure, Wanfang Data, China Biology Medicine Disc, China Science and Technology Journal Database, PubMed, and Web of Science). All statistical analyses were performed using STATA V.16.0.

**Results:**

Out of the 849 references identified, 26 articles were were eligible for inclusion, of which 12 reported the overall 12-month prevalence of WMSDs, while 14 stated the 12-month prevalence of WMSDs on body regions. The overall 12-month prevalence rate of WMSDs among workers was 53.1% [95% Confidence Interval (CI) = 46.3% to 59.9%]. The lower back/waist was the body region affected most (36.5%, 95%CI = 28.5% to 44.5%). The definition on WMSDs of “Chinese version” resulted in a high prevalence of WMSDs. Obesity, high educational level, long job tenure, female, logistic workers, and foundry workers are factors that led to a high prevalence rate of WMSDs in the lower back/waist.

**Conclusions:**

This study showed a high prevalence rate of WMSDs among auto workers in China. Thus, it is necessary to pay particular stress to them. Several effective measures should be taken to prevent these workers from WMSDs.

**Trial registration:**

This review was registered on PROSPERO (registration number CRD42023467152).

**Supplementary Information:**

The online version contains supplementary material available at 10.1186/s12889-023-16896-x.

## Introduction

Work-related musculoskeletal disorders (WMSDs) are the injuries and disorders of muscles, nerves, tendons, ligaments, joints, cartilage, spinal discs, and other skeletal system caused by occupational activities. The typical symptoms of WMSDs are discomfort, numbness, pain, and limited mobility in the affected areas [1]. WMSDs are associated with several occupational hazards and ergonomics factors, including mechanical workloads, awkward posture, repetitive action, vibration etc. In addition, psychosocial factors and individual factors are indispensable for the occurrence of WMSDs [2].

WMSDs have become a major occupational health problem in developed and developing country. In 2016, the occupational risk have led to 76.1 million disability cases worldwide, of which 20.3% cases were caused by occupational ergonomic factors (OEF). In 2019, musculoskeletal disorders (MSDs) attributable to OEF resulted in 5.5 million Disability-Adjusted Life Years (DALYs) among the youth population globally [3, 4]. For the years 1992–2010, MSDs accounted for 29–35% of all occupational injuries and diseases involving days away from work in the United States [5]. In 2019, more than 50 percent of employees working in the manufacturing industries of Europe were absent from work due to WMSDs, which was much more significant than those flu-related absences. In 60 percent of all reported cases of occupational diseases, WMSDs were the cause of permanent loss of workers' ability to work and live [6]. In Japan, MSDs are the single largest category of work-related illness, representing a third or more of all registered occupational diseases. Besides, medical costs of work-related low back pain were 82.14 billion yen, which is still on the increase [7, 8]. In Korea, WMSDs were about 70% of the among compensated occupational diseases, whose economic impacts have cost 7 billion dollars [9]. In China,WMSDs are widely distributed among various industries. Chinese Center for Disease Control and Prevention showed that the total standardized prevalence rate of WMSDs was 41.2% among the key industries for the past three years, such as electronic manufacturing industry, shoe-making industry, shipbuilding industry, etc. [10]. Furthermore, from 1990 to 2019, the average annual DALYs of WMSDs attributable to occupational risk factors were 203.80/100,000 [11].

Stimulated by economic and technological factors, automobile manufacturing industries have significantly and rapidly developed and become significant parts of modern manufacturing in China and other countries. Known for labor intensity, the automobile manufacturing industry imposes high physical exposure and poor ergonomic state on workers, which superposes workers at risk for musculoskeletal problems. Hence, it’s essential to protect these workers from WMSDs. For this reason, many countries pay great attention to WMSDs in this field. Various studies have been conducted to investigate the prevalence of WMSDs and the prevention of WMSDs among these workers [12–14]. However, WMSDs have not been incorporated into China’s legal occupational diseases list. Besides, many workers have to work by hand and withstand high workloads, although great strides have been made in automation and advanced equipment to minimize the risk of WMSDs. Despite numerous studies assessing the prevalence of WMSDs among workers in the Chinese automobile manufacturing industry, few meta-analyses have been published. Due to some considerations, such as the study area, object, sample size, etc., there were specific differences in prevalence among workers in various studies. Thus, to synthesize the results of previous studies, we conducted a systematic review and meta-analysis to discuss the prevalence of WMSDs among workers in the Chinese automobile manufacturing industry, generating the predictive intervals on WMSDs prevalence for future studies and providing a scientific basis for the prevention of WMSDs.

## Subjects and methods

### Search strategy

This study was carried out using the Systematic Reviews and Meta-analyses method under the most up to date PRISMA guidelines [15] (Details were shown in Table S1). A literature search was conducted for studies on the epidemiology of WMSDs among auto workers in China from inception to August 2022, using English and Chinese databases (China National Knowledge Infrastructure, Wanfang Data, China Biology Medicine Disc, China Science and Technology Journal Database, PubMed, and Web of Science). The keywords “musculoskeletal disorders”, “musculoskeletal pain”, “musculoskeletal injury”, etc., were used to searched the studies. No limitations to the language were imposed. The full search term string of each databases was shown in Table S 2.

### Criteria for considering studies

#### Inclusion criteria

Several criteria for included studies were defined in line with the Population, Exposure, Outcome, and Study Design (PEOS) framework: (i) Study participants were the front-line workers in the Chinese automobile manufacturing industry, such as welders, assembly workers, stamping workers, logistics workers, etc., except for office workers. (ii) The participants were exposed to the working conditions of automobile manufacturing. (iii) The outcome of the studies was musculoskeletal disorders. (iv) The studies provided information about the prevalence of WMSDs. (v) The studies were observational (cross-sectional, cohort, and case–control) or experimental study design.

#### Exclusion criteria

Studies were excluded if they: (i) were not accessible for a full review or provided insufficient information in their abstracts to calculate the prevalence; (ii) investigated outside China; (iii) failed to provide any prevalence of WMSDs in Chinese automobile manufacturing worker groups; (iv) were systematic review and meta-analysis; (v) were the studies carrying out on the same population.

### Selection of articles

The initial selection and screening of the articles against the inclusion and exclusion criteria were conducted by two authors independently. Disagreements were discussed with third reviewer and resolved in a consensus meeting. The articles were re-screened from preliminary screening to determine the ones that needed investigation.

### Methodological quality assessment and data extraction

#### Methodological quality assessment

The quality assessment was performed with an 11-item checklist which was recommended by Agency for Healthcare Research and Quality (AHRQ). An item would be scored ‘0’ if it was answered ‘NO’ or ‘UNCLEAR’; if it was answered ‘YES’, then the item scored ‘1’. A score less than or equal to 3 is classified as low quality, 4 to 7 is classified as moderate quality, and 8 to 11 is classified as high quality.

#### Data extraction

Two authors extracted the data independently to ensure the accuracy. Disagreements were discussed with third reviewer and resolved in a consensus meeting. The data and content of the articles contained the first author, publication time, study design, sample size, general characteristics, the criteria of WMSDs, the tools for data collecting and the rate of WMSDs. The rate of WMSDs on nine body regions(neck, shoulder, upper back, lower back/waist, elbow, wrist/hand, buttocks/leg, knee, ankle/foot) is recorded if possible. The prevalence rate and incidence rate of WMSDs are the most significant data of this systematic review and meta-analysis, which is the effective index.

## Statistical analysis

All analyses were performed using STATA V.16.0. Cochran Q test and I^2^ index were used to assess the heterogeneity of prevalence estimates among studies. For the Cochran Q test, *P* < 0.05 represented significant heterogeneity. For the I^2^ index, values of 50% or greater correspond to high degrees of heterogeneity, while values lower than 50% compare to low degrees of heterogeneity. A random-effects meta-analysis was used to calculate the pooled overall prevalence of WMSDs with 95% Confidence Interval (CI) throughout this study if heterogeneity was high; otherwise, a fixed-effects meta-analysis was used. The forest plot was used to represent the integrated results. To explore possible causes of heterogeneity among study results, specific subgroup analyses were planned to analyze the prevalence values for general characteristics like criteria of WMSDs, gender, job tenure, educational level, body mass index (BMI), profession, etc. The publication bias tested by Egger regression test.

## Results

### Selected articles

**Fig. 1:**
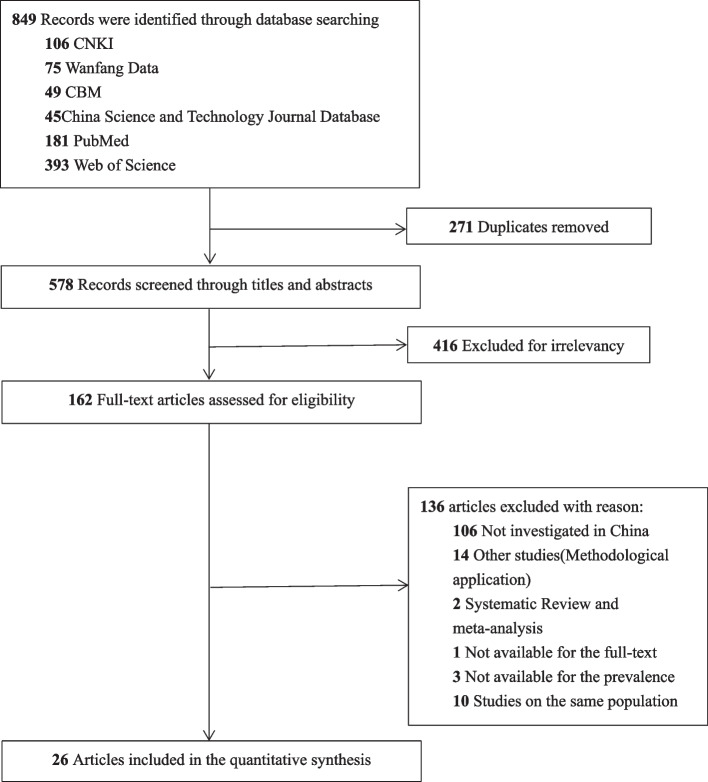
Flowchart of the process of literature search and selection

As outlined in Fig. 1, the initial search results of all databases were 849 articles. After removing duplicates by Note Express 3.4, 578 articles were screened, of which 271 were selected for evaluation on screening titles and abstracts. After this step, 26 articles [16–42] were included in the final assessment on a full-text base, of which 12 articles [16–27] reported the overall 12-month prevalence of WMSDs (Table 1) and the prevalence of WMSDs on body regions, while 14 articles [28–42] only provided the latter (Table 2). There were more than 37,184 automobile manufacturing workers in China analyzed. Although all the articles were Chinese, most of them represented the abstract in both Chinese and English expect for two articles, which only stated the abstract in Chinese [13, 30].

**Table 1:** General characteristics of 12 articles that reported the total annual prevalence of WMSDs

Study and Year	Sample size	Criterion For WMSDs	Tool For Survey	Incidence	Annual Prevalence	Annual Prevalence of body parts								
						Neck	Shoulder	Upper back	Lower back/Waist	Elbow	Wrist/Hand	Buttocks/Leg	Knee	Ankle/Feet
Liu X,2022 [16]	399	Chinese version	CMQ	181	45.4%	24.1%	17.5%	15.8%	16.5%	10.5%	17.0%	13.0%	13.0%	20.1%
Kang F,2019 [17]	663	Chinese version	CMQ	344	51.9%	29.7%	25.8%	17.0%	19.3%	9.7%	25.3%	25.3%	14.6%	22.6%
Cao L,2020 [18]	523	NIOSH	CMQ	283	54.1%	29.6%	25.8%	20.1%	15.5%	10.9%	13.9%	14.3%	16.8%	27.7%
Yang F,2020 [19]	554	NIOSH	CMQ	366	66.1%	39.5%	34.7%	26.4%	34.8%	11.4%	23.1%	17.1%	24.4%	33.9%
Wang H,2016 [20]	498	Self-definition	CMQ	418	84.0%	50.6%	50.6%	20.0%	43.4%	22.5%	38.4%	8.8%	20.2%	31.2%
Wang S,2019 [21]	839	Chinese version	CMQ	364	43.4%	24.7%	17.6%	15.0%	24.3%	4.6%	14.4%	4.2%	10.4%	12.0%
Wang Z,2017 [22]	1,494	Self-definition	NMQ	426	28.5%	13.1%	10.5%	3.7%	14.0%	4.4%	8.7%	8.2%	-	7.0%
Luo H,2022 [23]	264	NIOSH	CMQ	185	70.1%	39.0%	40.2%	33.3%	41.3%	18.9%	29.9%	26.9%	32.2%	30.7%
Shu Y,2021 [24]	831	NIOSH	CMQ	390	46.9%	30.7%	23.2%	15.3%	16.5%	5.3%	10.1%	10.5%	12.8%	20.2%
Chen P,2020 [25]	8,356	NIOSH	CMQ	3,727	44.6%	25.4%	21.4%	14.8%	16.8%	6.4%	13.7%	10.0%	13.0%	20.5%
Chen P,2021 [26]	7,065	NIOSH	CMQ	3,102	43.9%	24.5%	21.1%	14.5%	16.7%	6.4%	13.9%	9.8%	12.7%	20.1%
Ling R,2010 [27]	1,340	Self-definition	CMQ	789	58.9%	54.0%	46.3%	37.5%	58.9%	15.8%	32.2%	15.6%	28.8%	22.1%
Total	22,826			10,575										

*NIOSH* National Institute for Occupational Safety and Health, *CMQ* China Musculoskeletal Questionnaire, *NMQ* Nordic Musculoskeletal Questionnaire

**Table 2:** General characteristics of 14 articles that reported the annual prevalence of WMSDs in different body regions

Study and Year	Sample size	Criterion For WMSDs	Tool For Survey	Annual Prevalence of body parts								
				Neck	Shoulder	Upper back	Lower back/Waist	Elbow	Wrist/Hand	Buttocks/Leg	Knee	Ankle/Feet
Wu L, 2012 [28]	794	Self-definition	NMQ	-	-	-	47.0%	-	41.3%	-	-	-
Li Y, 2015 [29]	1465	Self-definition	NMQ	6.3%	4.9%	-	9.7%	-	6.5%	-	-	-
Jia N, 2017 [30]	184	Chinese version	CMQ	64.1%	51.6%	-	-	-	48.9%	25.5%	-	-
Liao H, 2020 [31]	808	Self-definition	CMQ	33.0%	24.9%	19.3%	32.3%	6.1%	21.2%	5.9%	13.2%	16.8%
Xu Y, 2020 [32]	484	NIOSH	CMQ	25.2%	18.6%	14.3%	16.9%	-	15.7%	12.0%	12.4%	18.4%
Wu J, 2013 [33]	1566	Chinese version	NMQ	57.4%	49.4%	45.0%	66.5%	25.0%	46.2%	22.4%	34.5%	28.8%
Fan Z, 1995 [34]	419	-	NMQ	34.4%	35.8%	35.8%	57.8%	28.4%	46.1%	15.8%	24.7%	22.2%
Chen S, 2018 [35]	394	Definition for LBP	CMQ	-	-	-	28.9%	-	-	-	-	-
Liu H, 1999 [36]	285	-	NMQ	37.9%	42.1%	45.9%	77.9%	-	51.2%	-	-	-
Wang S, 2018 [37]	400	Self-definition	Self-design	34.8%	-	-	-	-	-	-	-	-
Zhang K, 2020 [38]	754	Self-definition	CMQ	-	46.3%	-	65.9%	20.0%	-	-	-	-
Yao Y, 2022 [39]	677	Chinese version	CMQ	54.8%	-	-	-	-	-	-	-	-
Sun J, 2011 [40]	1290	-	CMQ	-	-	-	58.9%	-	-	-	-	-
Wu J, 2014 [41]	3800	Self-definition	CMQ	50.3%	42.7%	35.3%	60.3%	12.3%	30.8%	12.2%	25.5%	19.2%
Zhou H, 2011 [42]	1065	Self-definition	NMQ	47.5%	43.0%	61.8%	-	-	40.8%	-	-	-
Total	14,385											

*NIOSH* National Institute for Occupational Safety and Health, *CMQ* China Musculoskeletal Questionnaire, *NMQ* Nordic Musculoskeletal Questionnaire

### Quality of articles

Only AHRQ was used to assess the articles because all the included articles are cross-sectional study designs. According to the AHRQ, only one article was evaluated as high quality, 23 were considered medium quality, and two were assessed as low quality (Table 3).

**Table 3:** Quality assessment scores of cross-sectional studies (using the AHRQ scale)

Study	Source	Inclusion and exclusion criteria	Period of identifying patients	Consecutive subjects	Cover other information about the subjects	Quality control for outcome	Reasons for excluding analysis of some subjects	Evaluation and/or control of confounding	Handling lost data	Patient response rate and data integrity	Following	Total
Liu X,2022 [16]	1	1	1	0	1	0	0	1	0	1	0	6
Kang F,2019 [17]	1	1	1	0	1	1	1	1	0	1	0	8
Cao L, 2020 [18]	1	1	0	0	1	1	1	1	0	1	0	7
Yang F, 2020 [19]	1	1	0	0	1	1	0	1	0	1	0	6
Wang H, 2016 [20]	1	1	0	0	1	1	1	1	0	1	0	7
Wang S, 2019 [21]	1	1	1	0	1	1	0	1	0	1	0	7
Wang Z, 2017 [22]	1	0	0	0	1	1	1	1	0	1	0	6
Luo H, 2022 [23]	1	0	1	0	1	1	1	1	0	1	0	7
Shu Y, 2021 [24]	1	1	1	0	1	1	0	1	0	1	0	7
Chen P, 2020 [25]	1	1	1	0	1	1	0	1	0	1	0	7
Chen P, 2021 [26]	1	1	1	0	1	1	0	1	0	1	0	7
Ling R, 2010 [27]	1	1	0	0	1	1	0	1	0	1	0	6
Wu L, 2012 [28]	1	0	0	0	1	1	0	1	0	1	0	5
Li Y, 2015 [29]	1	0	0	0	1	1	1	1	0	1	0	6
Jia N, 2017 [30]	1	1	0	0	1	1	0	1	0	1	0	6
Liao H, 2020 [31]	1	1	1	0	1	1	0	1	0	1	0	7
Xu Y, 2020 [32]	1	1	0	0	1	0	0	1	0	1	0	5
Wu J, 2014 [33]	1	1	1	0	1	1	0	1	0	1	0	7
Fan Z, 1995 [34]	1	0	0	0	1	0	0	0	0	1	0	3
Chen S, 2018 [35]	1	1	0	0	1	0	0	1	0	1	0	5
Liu H, 1999 [36]	1	0	0	0	1	0	0	0	0	1	0	3
Wang S, 2018 [37]	1	1	1	0	1	0	0	1	0	1	0	6
Zhang K, 2020 [38]	1	1	1	0	1	1	0	1	0	1	0	7
Yao Y, 2022 [39]	1	1	1	0	1	1	0	1	0	1	0	7
Sun J, 2011 [40]	1	0	0	0	1	1	1	1	0	1	0	6
Wu J, 2013 [41]	1	0	0	0	1	1	0	1	0	1	0	5
Zhou H, 2011 [42]	1	0	0	0	1	1	0	1	0	1	0	5

“1” for “Yes”, “0” for “No” or “Not Clear”

### Meta-analysis on the prevalence of WMSDs among workers in the Chinese automobile manufacturing industry

#### The prevalence of WMSDs

**Fig. 2:**
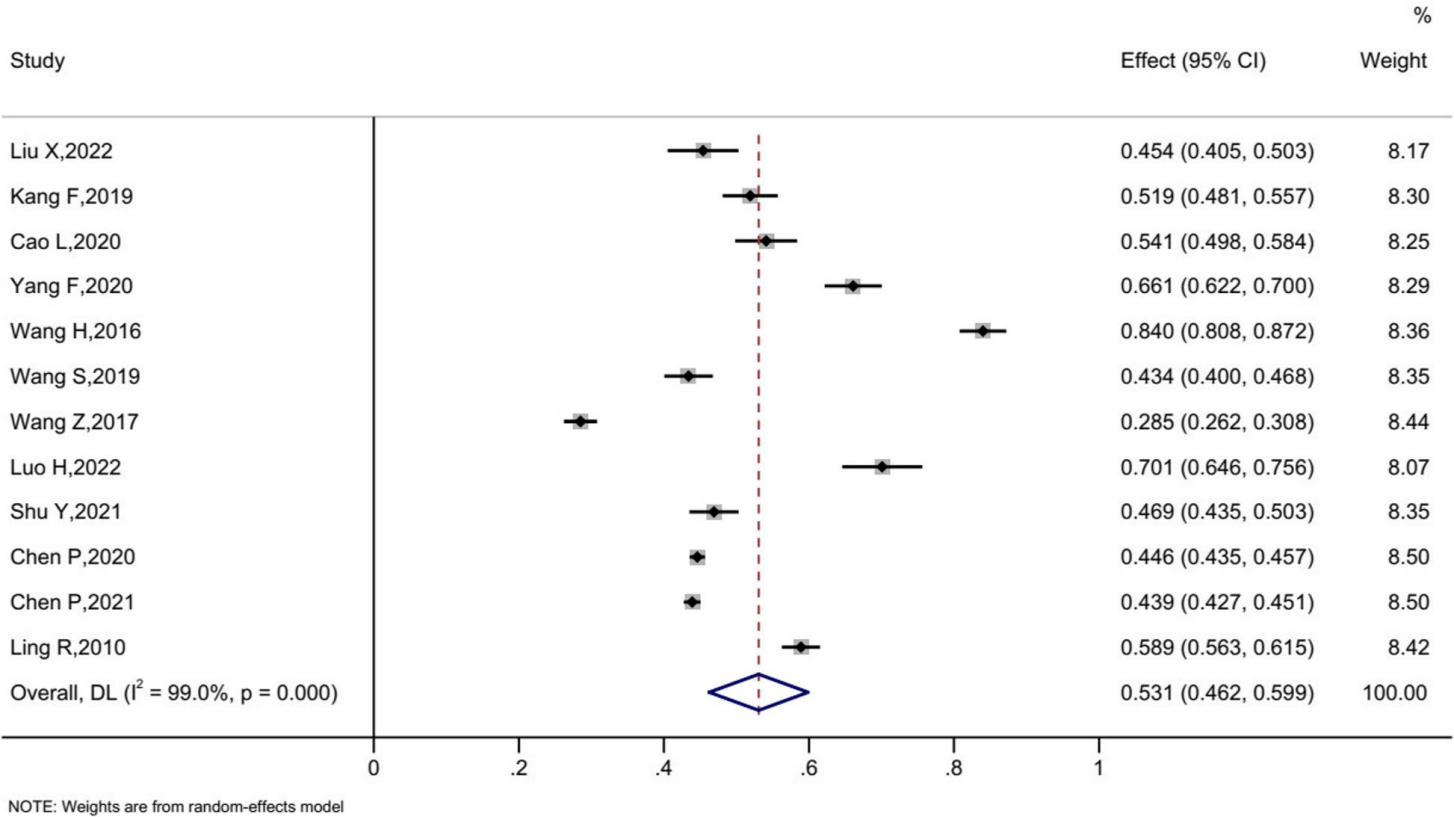
The overall 12-month prevalence of WMSDs among workers in Chinese automobile manufacturing

Twenty Six studies were eligible for the quantitative synthesis. There was evidence of significant heterogeneity in the meta-analysis, for which the random-effects model was used to calculate the combined effect value. The overall 12-month prevalence of WMSDs was 53.1% (95%CI = 46.3% to 59.9%). Stratified by body regions, the lower back/waist was affected most (36.5%, 95%CI = 28.5% to 44.5%). The prevalence of WMSDs in other body regions was following: 36.0% for neck (95%CI = 29.7% to 42.4%), 31.4% for shoulder (95%CI = 25.7% to 37.1%), 25.7% for upper back (95%CI = 20.0% to 31.5%), 12.5% for elbow (95%CI = 10.2% to 14.8%), 26.6% for wrist/hand (95%CI = 22.0% to 31.1%), 13.8% for buttocks/leg (95%CI = 11.8% to 15.8%), 19.2% for knee (95%CI = 15.9% to 22.5%) and 21.8% for ankle/feet (95%CI = 18.8% to 24.7%). All the stratification was extremely high heterogeneity (I^2^ > 90%, *P* < 0.05). Details are shown in Fig. 2 and Figure S 1. The result of Egger regression test (*p* = 0.123) proved that no publication bias existed.

#### The subgroup analysis

Given the significant variation in WMSDs outcomes based on criteria, a subgroup analysis for the overall prevalence of WMSDs was performed (Figure S 2). There are three criteria for WMSDs outcomes in these studies.

1. Chinese version. Based on the reality of Chinese workers and China Musculoskeletal Questionnaire (CMQ), the most commonly used definition of WMSDs in China is as follows: the symptoms like discomfort, numbness, pain, and limited mobility in the affected areas persist for more than 24 h and cannot be relieved after taking a break from work as well within the past 12 months [43].
2. National Institute for Occupational Safety and Health (NIOSH). According to NIOSH, WMSDs are defined as follows: (1) discomfort within the past year; (2) discomfort beginning after employment in the current job; (3) no prior accident or sudden injury (affecting a focal area of discomfort); and (4) episodes of discomfort occurring monthly or, if not every month, at least exceeding a week-long period of discomfort [44].
3. Self-definition. The author defines WMSDs by references. These definitions are diverse.

The overall 12-month prevalence of WMSDs based on definition of Chinese version was 46.9% (95%CI = 41.5% to 52.3%). The overall 12-month prevalence of WMSDs based on definition of NIOSH was 53.8% (95%CI = 48.2% to 59.4%). The overall 12-month prevalence of WMSDs based on self-definition was 57.1% (95%CI = 25.9% to 88.3%).

What’s more, considering the limited number of studies that included automobile manufacturing workers’ characteristics and the high prevalence of WMSDs on the lower back/waist, it was possible to perform a subgroup analysis for the lower back/waist region only (Figure S 3).

##### Gender

The prevalence of WMSDs on the lower back/waist in females was 61.0% (95%CI = 49.8% to 72.1%) while it was 54.7% (95%CI = 36.1% to 73.2%) in males.

##### Job tenure

Among workers with long job tenure (more than five years), the prevalence was 46.0% (95%CI = 20.9% to 71.1%), and it was 36.7% (95%CI = 16.3% to 57.2%) in workers with short job tenure (below or equal to five years).

##### Educational level

For workers with low educational level (senior high or below), the prevalence was 42.5% (95%CI = 27.3% to 57.7%); for workers with high educational level (undergraduate or above), it was 47.0% (95%CI = 30.5% to 63.5%).

##### BMI

The prevalence of workers in low BMI (below 18.5 kg/m^2^) was 43.3% (95%CI = 22.9% to 63.7%), while the group in average BMI (range from 18.5 to 23.9 kg/m^2)^ was 47.2% (95%CI = 29.8% to 64.6%) and the population in high BMI (over 24 kg/m^2^) was 49.9% (95%CI = 31.6% to 68.3%).

##### Profession

In different workshops, the logistic and foundry workers had high rates of WMSDs to be 34.4% (95%CI = 16.0% to 52.8%) and 43.4% (95%CI = 21.7% to 65.1%).

## Discussion

In this study, we investigated the overall 12-month prevalence of WMSDs among workers in the Chinese automobile manufacturing industry ranged from 28.5% to 84.0%, which varied significantly. This variability can be attributed to the differences in the standard of criteria on WMSDs. A subgroup analysis for different criteria on WMSDs showed that the overall 12-month prevalence rate of WMSDs defined by “Self-definition” was the highest. Among the three definition of WMSDs, the definition on WMSDs of “Self-definition” was not rigorous, resulting in a higher incidence rate. On the contrary, the overall 12-month prevalence rate of WMSDs defined by “Chinese version” was the lowest because this definition is relatively strict, especially in the aspects of time. In these articles, Nordic Musculoskeletal Questionnaire (NMQ) and China Musculoskeletal Questionnaire (CMQ) were mainly used for investigating the prevalence of WMSDs. Although CMQ is adapted from NMQ, there were no significant difference in functions between CMQ and NMQ when using for prevalence survey only [45–47].

Automobile manufacturing workers in China bear a relatively huge burden of WMSDs. The overall 12-month prevalence estimate of WMSDs among these workers was 53.1% (95%CI = 46.3% to 59.9%). However, this result is inconsistent with the rate reported in Korea, India, Malaysia and other countries. 27.4% Workers in the automobile sector in Korea complaint that they have musculoskeletal pain in any one area [48]. Among automobile repair workers in India, 85% of them reported pain in different body regions while 87% workers complaint that they have suffered from multi-site pain in the past year [49]. In Malaysia, 91.7% workers in a car tyre service centre have body discomfort in the hand/wrist. In a metal autoparts factory in eastern Thailand, almost all the employees felt discomfort on musculoskeletal system [50]. This may be due to the different working conditions in different countries. Workers in developed countries utilize more automatic machines and advanced technologies than in developing countries. Thus, workers do less by hand. Compared with specific manufacturing industries, the prevalence was relatively high. Among electronics manufacturing workers, the overall 12-month prevalence of musculoskeletal symptoms was 40.6% [51]. This may be because automobile manufacturing needs a high physical load. Nevertheless, the prevalence was relatively low compared to professions requiring manual operation in most cases. For instance, a systematic review and meta-analysis reported a high-quality article for dentists writing an extremely high prevalence (97.9%) [52]. This may be related to the application of automation and advanced equipment in the automobile manufacturing industry, which could significantly minimize the manual operation of automobile manufacturing workers to some extent and reduce the possibility of musculoskeletal system obstacles and injuries. Apart from the impact of occupational characteristics, the individual susceptibility to WMSDs also plays a role in these discrepancies. The genetic factors make individuals more vulnerable to MSDs through their resultant contribution to both physical structure and chemical environments [53].

It is worth noting that the the upper parts of the body was the most affected body region. This was consistent with some studies’ findings [54–58]. Automobile manufacturing needs workers to operate with the upper parts of the body frequently, such as stamping, painting, and assembling. Thus, WMSDs occur in the relevant body parts like the neck, shoulder, lower back/waist, and elbow. Besides, these workers have to maintain awkward postures frequently and chronically in the lower back/waist, like turning around repeatedly, bending for a long time, leaning by a wide margin, etc. These poor postures made workers feel fatigued without recovery, increasing the risk of WMSDs on the lower back/waist.

Several personal characteristics could be related to WMSDs, such as gender, job tenure, educational level, body mass index (BMI), etc. The female workers seemed to be related to WMSDs on the lower back/waist. The most likely explanation for the increased rate of female workers might be the gender differences in somatic, hormonal, and psychological aspects [59]. The structure of the female’s muscle and ligament soft tissue on the lower back/waist are relatively weak compared with males. In work requiring object transfer, even the experienced female workers had to choose the analogous posture to that of novices when spine loading was critical. In other words, these female workers withstood a high spine load, and the lumbar musculoskeletal system of these female workers was at increased risk of injury [60]. This situation worsens in the automobile manufacturing industry, known for its labor intensity. There is another reason to note that most women are usually responsible for housework even after hard work. Some housework requires women to walk or bend for long periods and remain to stand, aggravating the injury of the lower back/waist. These issues might quickly increase the load on the lumbar muscle of females, bring about lumbar muscle strain, reduce their rest time and induce the accumulation of fatigue.

According to a recent study, novices or younger workers seem more vulnerable to WMSDs. The experienced workers or/and elder workers were prone to select protective measures, such as seeking colleagues for help, taking a rest during work, etc., to minimize the risk of WMSDs [61]. However, this study found that WMSDs on the lower back/waist worsened with the increasing working years. This might be because the workers with high working ages were well-rounded in manufacturing. In other words, they have to pick up more shifts frequently and undertake complicated work, with the problems like a decrease in rest time, accumulation of fatigue, repetitive movement, etc. Besides, high working ages mean growth of age to some extent. Musculoskeletal mass and strength decline with age, leading to reduced tolerance to load and decreased ability to recover from fatigue.

Several studies showed that the prevalence of WMSDs in the occupational population decreased with the improvement of educational level. The people with an advanced degree, who knew more information about occupational health, protect themselves by taking the initiative. However, the result of the subgroup analysis was contrary to the above. Still, there was little variability of prevalence between the workers with low and high education backgrounds, which might cause by those with high educational levels who account for more in the automobile manufacturing industries.

Obesity is a common risk factor for musculoskeletal disorders. Research showed that the prevalence of musculoskeletal disorders in the obese population was higher than in the general population. Patrick Hiepe [62] noted that high-fat inclusion impaired lumbar muscle function with long-lasting and highly intense loads. Therefore, the automobile manufacturing workers with high BMI or obesity were prone to be suffered from WMSDs on the lower back/waist because their lower back/waist was inherently loaded when working.

There are different tasks in the automobile manufacturing industry, whereby these workers face other working conditions and working organization. Logistic and foundry workers must frequently bend, twist, lift weights, and stand for long periods [40]. Evidence shows that the heavy physical workload and accumulation of loads or frequency of lifts were moderate to vital risk factors for low back pain (LBP). Besides, bending and twisting are highly associated with LBP [63]. Dieter Coenen [64] also indicated that occupational standing for long periods was relevant to LBP.

In summary, WMSDs represented a high prevalence issue among workers in the automobile manufacturing industry in China. Thus, the managers should attach importance to the protection of workers. Although most personal characteristics could hardly be changed, there are still measures in other aspects to prevent workers from WMSDs. For example, the application of automated production and advanced equipment was one of the effective measures which could reduce manual operation and muscle load to a great extent and improve productivity as well. To some risk factors that could not be avoided by automation and advanced equipment, the upgradation of working organization and working conditions, such as increasing workforce, allocating work reasonably, job rotation, etc., might be other effective methods to reduce the workload and the time of maintaining poor postures and provide more time for workers to take a rest. Besides, ergonomics should be applied more to the working environment. Ergonomics work design combined with good team diversity might compensate for age-related productivity risks in automobile manufacturing by maintaining the working ability of older employees and improving job quality [65]. Furthermore, occupational health education is essential and plays a positive role in managing WMSDs [66], which should be regularly in progress to enhance the occupational health literacy of workers. What’s more, managers are suggested to conduct physical examinations for workers regularly, finding out the health problems of workers and taking precautions in time to reduce the risk of WMSDs.

However, this systematic review and meta-analysis had some potential limitations. First, the Grey Literature was omitted. Second, the kind of detailed studies was too narrow because all the studies involved in the review were cross-sectional studies. Third, due to the high heterogeneity within the meta-analyses, the results should be interpreted with care.

## Conclusion

The prevalence of WMSDs among workers in the automobile manufacturing industry in China appears to be high. These workers are at risk of WMSDs. The most affected body region was the lower back/waist, while the neck and shoulder regions were also easily influenced. The different definition on WMSDs resulted in different prevalence of WMSDs. Females, people with obesity, high educational background, and high working years might be the susceptible population of WMSDs on the lower back/ waist in this field. Moreover, logistics and foundry workers’ WMSDs on the lower back/ waist were notable. Some effective measures should be taken to prevent workers from WMSDs, like applying ergonomics, improving working conditions, and working environment. Furthermore, our study could help promote the inclusion of WMSDs in the statutory list of occupational diseases.

### Supplementary Information

**Additional file 1. **Preferred Reporting Items for Systematic Reviews and Meta-Analyses (PRISMA) 2020 Checklist.

**Additional file 2: Table S2**. The full search term string of each databases.

**Additional file 3: Table S3. **Agency for Healthcare Research and Quality (US).

**Additional file 4: Table S4.** PEOS (Population, Exposure, Outcome and Study Design) of each article.

**Additional file 5: Figure S1**. The prevalence of WMSDs in nine body regions among Chinese automobile manufacturing workers. The nine body regions referred to neck, shoulder, upper back, lower back/waist, elbow,wrist/hand, buttocks/leg, knee and ankle/feet. “Effect” referred to the prevalence rate.

**Additional file 6: Figure S2**. The overall 12-month prevalence of WMSDs based on different criteria. The criteria were “Chinese version”, “NIOSH” and “Self-definition” respectively. “Effect” referred to the prevalence rate.

**Additional file 7: Figure S3.** The prevalence of WMSDs on the lower back/waist among Chinese automobile manufacturing workers by personal characteristics (Gender, Job tenure, BMI, Education level, Profession). “Effect” referred to the prevalence rate.

## Data Availability

All data generated or analysed during this study are included in this published article and its supplementary information files.

## Abbreviations

WMSDs: Work-related musculoskeletal disorders
MSDs: Musculoskeletal disorders
OEF: Occupational ergonomic factor
DALYs: Disability-Adjusted Life Years
BMI: Body mass index
AHRQ: Agency for Healthcare Research and Quality
