# Supplementary material for: Prevalence of work-related musculoskeletal disorders among workers in the automobile manufacturing industry in China: a systematic review and meta-analysis
Source: BMC Public Health. 2023 Oct 19;23:2042. doi: 10.1186/s12889-023-16896-x (PMC10585820; doi:10.1186/s12889-023-16896-x)
Supplement: Supplementary file 2 — Additional file 2: Table S2. The full search term string of each databases. [file 12889_2023_16896_MOESM2_ESM.docx]

**Table S2** The full search term string of each databases

| Database | Number of literature | Full search term string |
| --- | --- | --- |
| China National Knowledge Infrastructure | 106 | (SU=MSDs OR SU=WMSDs OR SU=OMDs OR SU=工作相关肌肉骨骼疾患 OR SU=职业性肌肉骨骼疾患 OR SU=肌肉骨骼疼痛 OR SU=肌肉骨骼损伤 OR SU=肌肉骨骼) AND (SU=汽车 OR SU=汽车制造) |
| Wanfang Data | 75 | ((主题:(MSDs) or 主题:(OMDs) or 主题:(WMSDs) or 主题:(工作相关肌肉骨骼疾患) or 主题:(职业性肌肉骨骼疾患) or 主题:(肌肉骨骼疼痛) or 主题:(肌肉骨骼损伤)) and ((主题:(汽车) or 主题:(汽车制造)) and Date:*-2022 |
| China Biology Medicine Disc | 49 | ("肌肉骨骼"[常用字段:智能] OR "肌肉骨骼疼痛"[常用字段:智能] OR "肌肉骨骼损伤"[常用字段:智能] OR "工作相关肌肉骨骼疾患"[常用字段:智能] OR "职业性肌肉骨骼疾患"[常用字段:智能]) AND ("汽车"[常用字段:智能] OR "汽车制造"[常用字段:智能]) |
| China Science and Technology Journal Database | 45 | (M=肌肉骨骼 OR M=肌肉骨骼疾患 OR M=肌肉骨骼损伤 OR M=工作相关肌肉骨骼疾患 OR M=职业性肌肉骨骼疾患) AND (M=汽车 OR M=汽车制造) |
| PubMed | 181 | ((musculoskeletal) OR (musculoskeletal disorders) OR (musculoskeletal pain) OR (pain) OR (musculoskeletal injury) OR (work-related musculoskeletal disorders) OR (occupational musculoskeletal disorders)) AND ((automobile industry) OR (automobile making)) AND (1000/1/1:2022/8/31[pdat]) |
| Web of Science | 393 | ((TS=musculoskeletal disorders) OR TS=(musculoskeletal injury) OR TS=(musculoskeletal) OR TS=(musculoskeletal pain) OR TS=(pain) OR TS=(work-related musculoskeletal disorders) OR TS=(occupational musculoskeletal disorders)) AND (TS=(automobile industry) OR TS=(automobile manufacturing) OR TS=(automobile making)) |
| Total | 849 |  |
