# Supplementary material for: Prevalence of work-related musculoskeletal disorders among workers in the automobile manufacturing industry in China: a systematic review and meta-analysis
Source: BMC Public Health. 2023 Oct 19;23:2042. doi: 10.1186/s12889-023-16896-x (PMC10585820; doi:10.1186/s12889-023-16896-x)
Supplement: Supplementary file 3 — Additional file 3: Table S3. Agency for Healthcare Research and Quality (US). [file 12889_2023_16896_MOESM3_ESM.docx]

**Table S3** Agency for Healthcare Research and Quality (US)

| Entry | Yes | No | Not clear |
| --- | --- | --- | --- |
| 1. Define the source of information (survey, record review) |  |  |  |
| 2. List inclusion and exclusion criteria for exposed and unexposed subjects (cases and controls) or refer to previous publications |  |  |  |
| 3. Indicate time period used for identifying patients |  |  |  |
| 4. Indicate whether or not subjects were consecutive if not population-based |  |  |  |
| 5. Indicate if evaluators of subjective components of study were masked to other aspects of the status of the participants |  |  |  |
| 6. Describe any assessments undertaken for quality assurance purposes (e.g., test/retest of primary outcome measurements) |  |  |  |
| 7. Explain any patient exclusions from analysis |  |  |  |
| 8.Describe how confounding was assessed and/or controlled. |  |  |  |
| 9. If applicable, explain how missing data were handled in the analysis |  |  |  |
| 10.Summarize patient response rats and completeness of data collection |  |  |  |
| 11.Clarify what follow-up, if any, was expected and the percentage of patients for which incomplete data or follow-up was obtained |  |  |  |
