# Supplementary material for: Prevalence of work-related musculoskeletal disorders among workers in the automobile manufacturing industry in China: a systematic review and meta-analysis
Source: BMC Public Health. 2023 Oct 19;23:2042. doi: 10.1186/s12889-023-16896-x (PMC10585820; doi:10.1186/s12889-023-16896-x)
Supplement: Supplementary file 4 — Additional file 4: Table S4. PEOS (Population, Exposure, Outcome and Study Design) of each article. [file 12889_2023_16896_MOESM4_ESM.docx]

Table S4 PEOS (Population, Exposure, Outcome and Study Design) of each article

| **Study** | **Population** | **Exposure** | **Outcome** | **Study Design** |
| --- | --- | --- | --- | --- |
| Liu X,2022 | 399 front-line workers in an automobile manufacturing company | Working in automobile manufacturing over one year | The overall 12-month prevalence of WMSDs and the prevalence of WMSDs in body regions | cross-section study |
| Kang F,2019 | 663 automobile assembly male workers in Shandong Province | Working in automobile manufacturing | The overall 12-month prevalence of WMSDs and the prevalence of WMSDs in body regions | cross-section study |
| Cao L,2020 | 523 male automobile workers in Chongqing city | Working in automobile manufacturing over one year | The overall 12-month prevalence of WMSDs and the prevalence of WMSDs in body regions | cross-section study |
| Yang F,2020 | 554 workers in auto manufacturing factory | Working in automobile manufacturing over one year | The overall 12-month prevalence of WMSDs and the prevalence of WMSDs in body regions | cross-section study |
| Wang H,2016 | 498 male automobile  assembling workers | Working in automobile manufacturing | The overall 12-month prevalence of WMSDs and the prevalence of WMSDs in body regions | cross-section study |
| Wang S,2019 | 839 automobile manufacturing industry workers | Working in automobile manufacturing | The overall 12-month prevalence of WMSDs and the prevalence of WMSDs in body regions | cross-section study |
| Wang Z,2017 | 1494 male workers in automobile manufacturing  industry | Working in automobile manufacturing over one year | The overall 12-month prevalence of WMSDs and the prevalence of WMSDs in body regions | cross-section study |
| Luo H,2022 | 264 workers in heavy-duty automobile parts factories | Working in automobile manufacturing over one year | The overall 12-month prevalence of WMSDs and the prevalence of WMSDs in body regions | cross-section study |
| Shu Y,2021 | 831 workers in an auto parts manufacturing enterprise | Working in automobile manufacturing over one year | The overall 12-month prevalence of WMSDs and the prevalence of WMSDs in body regions | cross-section study |
| Chen P,2020 | 8,356 workers from a vehicle manufacturing enter prise and an auto parts manufacturer in Guangzhou city | Working in automobile manufacturing | The overall 12-month prevalence of WMSDs and the prevalence of WMSDs in body regions | cross-section study |
| Chen P,2021 | 7065 workers of an automobile manufacturing enterprise in Guangzhou City | Working in automobile manufacturing over one year | The overall 12-month prevalence of WMSDs and the prevalence of WMSDs in body regions | cross-section study |
| Ling R,2010 | 1,340 workers from an automobile foundry factory | Working in automobile manufacturing | The overall 12-month prevalence of WMSDs and the prevalence of WMSDs in body regions | cross-section study |
| Wu L, 2012 | 794 automobile manufacturing workers | Working in automobile manufacturing | The 12-month prevalence of WMSDs in body regions | cross-section study |
| Li Y, 2015 | 1,465 Automobile Assembly male Workers | Working in automobile manufacturing | The 12-month prevalence of WMSDs in body regions | cross-section study |
| Jia N, 2017 | 184 automobile  assembly male workers | Working in automobile manufacturing over one year | The 12-month prevalence of WMSDs in body regions | cross-section study |
| Liao H, 2020 | 808 front-line workers in an automobile manufacturing company | Working in automobile manufacturing over one year | The 12-month prevalence of WMSDs in body regions | cross-section study |
| Xu Y, 2020 | 484 workers in an automobile manufacturing enterprise | Working in automobile manufacturing over one year | The 12-month prevalence of WMSDs in body regions | cross-section study |
| Wu J, 2013 | 3800 auto workers | Working in automobile manufacturing | The 12-month prevalence of WMSDs in body regions | cross-section study |
| Fan Z, 1995 | 419 auto workers | Working in automobile manufacturing | The 12-month prevalence of WMSDs in body regions | cross-section study |
| Chen S, 2018 | 394 automobile factory workers | Working in automobile manufacturing over one year | The 12-month prevalence of WMSDs in body regions | cross-section study |
| Liu H, 1999 | 285 workers in an automobile factory | Working in automobile manufacturing | The 12-month prevalence of WMSDs in body regions | cross-section study |
| Wang S, 2018 | 446 workers in automobile plant | Working in automobile manufacturing over one year | The 12-month prevalence of WMSDs in body regions | cross-section study |
| Zhang K, 2020 | 754 electric  welders in an automobile factory | Working in automobile manufacturing over one year | The 12-month prevalence of WMSDs in body regions | cross-section study |
| Yao Y, 2022 | 677 electric welders from an automobile manufacturing plant in Shiyan City | Working in automobile manufacturing over one year | The 12-month prevalence of WMSDs in body regions | cross-section study |
| Sun J, 2011 | 1340 workers in a foundry factory of the automoble company | Working in automobile manufacturing | The 12-month prevalence of WMSDs in body regions | cross-section study |
| Wu J, 2014 | 1566 auto workers | Working in automobile manufacturing over one year | The 12-month prevalence of WMSDs in body regions | cross-section study |
| Zhou H, 2011 | 1065 workers from 3 automobile manufacturers in south China | Working in automobile manufacturing | The 12-month prevalence of WMSDs in body regions | cross-section study |
