# Supplementary material for: Prevalence of work-related musculoskeletal disorders among workers in the automobile manufacturing industry in China: a systematic review and meta-analysis
Source: BMC Public Health. 2023 Oct 19;23:2042. doi: 10.1186/s12889-023-16896-x (PMC10585820; doi:10.1186/s12889-023-16896-x)
Supplement: Supplementary file 5 — Additional file 5: Figure S1. The prevalence of WMSDs in nine body regions among Chinese automobile manufacturing workers. The nine body regions referred to neck, shoulder, upper back, lower back/waist, elbow,wrist/hand, buttocks/leg, knee and ankle/feet. “Effect” referred to the prevalence rate. [file 12889_2023_16896_MOESM5_ESM.docx]

**Figure S1 The prevalence of WMSDs in nine body regions among Chinese automobile manufacturing workers.** The nine body regions referred to neck, shoulder, upper back, lower back/waist, elbow, wrist/hand, buttocks/leg, knee and ankle/feet. “Effect” referred to the prevalence rate.
