# Supplementary material for: Prevalence of work-related musculoskeletal disorders among workers in the automobile manufacturing industry in China: a systematic review and meta-analysis
Source: BMC Public Health. 2023 Oct 19;23:2042. doi: 10.1186/s12889-023-16896-x (PMC10585820; doi:10.1186/s12889-023-16896-x)
Supplement: Supplementary file 6 — Additional file 6: Figure S2. The overall 12-month prevalence of WMSDs based on different criteria. The criteria were “Chinese version”, “NIOSH” and “Self-definition” respectively. “Effect” referred to the prevalence rate. [file 12889_2023_16896_MOESM6_ESM.docx]

**Figure S2** **The overall 12-month prevalence of WMSDs based on different criteria.** The criteria were “Chinese version”, “NIOSH” and “Self-definition” respectively. “Effect” referred to the prevalence rate.
