# Supplementary material for: Prevalence of work-related musculoskeletal disorders among workers in the automobile manufacturing industry in China: a systematic review and meta-analysis
Source: BMC Public Health. 2023 Oct 19;23:2042. doi: 10.1186/s12889-023-16896-x (PMC10585820; doi:10.1186/s12889-023-16896-x)
Supplement: Supplementary file 7 — Additional file 7: Figure S3. The prevalence of WMSDs on the lower back/waist among Chinese automobile manufacturing workers by personal characteristics (Gender, Job tenure, BMI, Education level, Profession). “Effect” referred to the prevalence rate. [file 12889_2023_16896_MOESM7_ESM.docx]

**Figure S3 The prevalence of WMSDs on the lower back/waist among Chinese automobile manufacturing workers by personal characteristics (Gender, Job tenure, BMI, Education level, Profession).**  “Effect” referred to the prevalence rate.
